# Supplementary material for: Evaluation of Green Super Rice Lines for Agronomic and Physiological Traits under Salinity Stress
Source: Plants (Basel). 2022 May 30;11(11):1461. doi: 10.3390/plants11111461 (PMC9182741; doi:10.3390/plants11111461)
Supplement: Supplementary file 1 [file plants-11-01461-s001.zip › plants-1723174-supplementary.pdf]

Supplementary Table S1. Twenty-four rice genotypes used for this study.

Genotype Name	Code	Country of Origin
GSR-434	S1	China
GSR-48	S2	China
GSR-305	S3	China
GSR-139	S4	China
GSR-130	S5	China
GSR-127	S6	China
GSR-112	S7	China
GSR-52	S8	China
GSR-268	S9	China
GSR-521	S10	China
GSR-63	S11	China
GSR-147	S12	China
GSR-54	S13	China
GSR-69	S14	China
GSR-519	S15	China
GSR-244	S16	China
GSR-116	S17	China
GSR-119	S18	China
GSR-249	S19	China
GSR-510	S20	China
GSR-82	S21	China
GSR-252	S22	China
IRRI6 (Check)	S23	Philippines
Kissan Basmati (Check)	S24	Pakistan

Supplementary Table S2. Soil characteristics of the site used for field evaluation.

Soil Sample	AB-DPTA (mg kg^{-1})			1:1 Ratio		
	**K**	**NO_3-N**	**P**	**EC (dSm-^{1})**	**SAR**	**pH**
Control R1	94	2.11	19.48	0.22	0.86	7.38
Control R2	68	1.16	11.16	0.21	0.76	7.58
Control R3	70	1.11	13.64	0.21	1.65	7.61
Salt R1	252	0.85	7.96	0.83	2.76	7.76
Salt R2	270	2.13	9.39	0.69	1.04	8.49
Salt R3	282	3.11	15.82	1.33	1.07	9.03

Under field conditions three replicates of control and Salt stress; **K** = potassium; **NO_3-N** = nitrate-nitrogen; **P** = phosphorus; **EC** = Electrical conductivity; **SAR** = sodium adsorption ratio

Supplementary Table S3. Primers sequences used for real-time PCR analysis.

Primer name		Primer sequence (5′-3′)
LOC_Os08g34540	F	AATCACGCCATGAAGGGAGACTAC
	R	TCTTTTGCCTGGGAGAACCACTTG
LOC_Os05g33260	F	CATCGTGACTGACAGATGGCAGAA
	R	ACTCCCAACCGTAACATCAACTCG

LOC_Os03g43850	F	TGCTGAGCTGATGGAAGTCGTT
	R	CTTGCTCCTTCTGGTATCATGGCT
LOC_Os05g04830	F	CTGCACCGTGGAGATACAATGCTA
	R	CGCCTTCACATCCATCGGATCAT
LOC_Os01g04950	F	TGTTCTTGGCGCTGTACCTGATAG
	R	CGCCGATGTTGATGGAGAAGTAGA
LOC_Os01g20160 (SKC1/OsHKT8)	F	AACTACAGCGTCCTCAACATCGTC
	R	TGAGAGTGAGCTTCCCTTGTTTGC
LOC_Os03g37930 (qSE3/OsHAK21)	F	CATGCCGAGGATATGCTTACACCA
	R	GAAGCGATGAGTGAGGCCTTACAA
LOC_Os02g52780 (OsbZIP23)	F	GTGCCGTACGTTTTCAAAGGTG
	R	GCTACCTCAGCTTCCAATTCCATC
OsActin	F	TCCATCTTGGCATCTCTCAG
	R	GGTACCCTCATCAGGCATCT

Supplementary Table S4. Mean summary statistics of 12 morpho-physiological parameters at early seedling stage of 24 rice genotypes.

Parameters	Treatment	Maximum	Minimum	Mean	SD	Reduction (%)
SL	Control	29.83	24.16	26.43	1.57	5.65
	Salinity	29.91	20.34	24.94	2.03	
RL	Control	23.01	16.99	20.88	1.35	11.20
	Salinity	21.16	14.81	18.54	1.57	
TPL	Control	51.56	42.92	47.31	2.24	8.10
	Salinity	49.35	36.86	43.48	2.95	
SFW	Control	0.23	0.12	0.19	0.02	38.39
	Salinity	0.15	0.08	0.11	0.01	
RFW	Control	0.20	0.12	0.16	0.01	45.66
	Salinity	0.12	0.06	0.09	0.01	
SDW	Control	0.06	0.03	0.05	0.00	33.75
	Salinity	0.04	0.02	0.03	0.00	
RDW	Control	2.69	0.02	0.14	0.54	22.63
	Salinity	2.23	0.01	0.10	0.45	
SNC	Control	5	1.13	1.95	0.74	-219.42
	Salinity	9.13	2.93	6.25	1.72	
SKC	Control	9.36	4.9	5.67	0.84	30.54
	Salinity	6.06	2.43	3.94	0.99	
RNC	Control	1.83	0.23	0.63	0.29	-102.46
	Salinity	1.70	0.85	1.29	0.24	
RKC	Control	2.51	0.78	1.14	0.33	-34.10
	Salinity	2.98	0.85	1.53	0.42	
NKR	Control	0.83	0.25	0.38	0.11	-270.64
	Salinity	2.34	0.76	1.42	0.40	

SD = standard deviation; **SL** = shoot length (cm); **RL** = root length (cm); **TPL** = total plant length (cm); **SFW** = shoot fresh weight (g); **RFW** = root fresh weight (g); **SDW** = shoot dry weight (g); **RDW** = root dry weight (g); **SNC** = shoot sodium (Na^+) concentration; **SKC** = shoot potassium (K^+) concentration; **RNC** = root Na^+ concentration; **RKC** = root K^+ concentration; **NKR** = whole plant sodium/potassium (Na^+/K^+) ratio.

Supplementary Table S5. Mean summary statistics of seven morpho-physiological parameters at maturity of 24 rice genotypes.

Parameters	Treatment	Maximum	Minimum	Mean	SD	Reduction (%)
**PH**	**Control**	114.13	90.93	100.53	6.79	0.92
	**Salinity**	117.53	88.33	99.60	7.26	
**TN**	**Control**	29.46	19.73	24.78	2.55	27.02
	**Salinity**	22.2	14.33	18.9	2.51	
**GY**	**Control**	107.6	64.48	91.27	11.61	59.16
	**Salinity**	51.46	23.93	37.27	6.76	
**SY**	**Control**	120.14	78.00	107.37	11.45	17.62
	**Salinity**	125.6	43.6	88.45	17.39	
**TGW**	**Control**	29.20	20.25	22.86	2.44	10.45
	**Salinity**	30.62	17.43	20.47	2.88	
**GL**	**Control**	12.54	7.94	9.43	0.87	-0.72
	**Salinity**	12.18	7.81	9.50	0.85	
**HI**	**Control**	0.47	0.42	0.45	0.015	33.57
	**Salinity**	0.41	0.15	0.30	0.05	

SD = standard deviation; **PH** = plant height (cm); **TN** = tillers number; **GY** = grain yield/plant (g); **SY** = straw yield (g); T**GW** = 1000-grain weight (g); **GL** = grain length (mm); **HI** = harvest index (%).
